# Clinical, genetic and environmental influences on weight gain and metabolic disorders induced by psychotropic drugs

**DOI:** 10.1192/j.eurpsy.2022.280

**Published:** 2022-09-01

**Authors:** C. Eap

**Affiliations:** Center for Psychiatric Neurosciences, Department Of Psychiatry, Prilly-Lausanne, Switzerland

**Keywords:** Genetics, metabolic syndrome, psychotropic drugs, epigenetics

## Abstract

**Introduction:**

Weight gain and obesity are important health problems associated with psychiatric disorders and/or with psychotropic drug treatments. There is a high inter-individual variability in the susceptibility to drug induced weight gain and/or other cardiometabolic disorders.

**Objectives:**

To study the genetic and environmental risk factors for weight gain and onset of metabolic syndrome during psychotropic treatment

**Methods:**

Analysis in PsyMetab, a large (n>3000) ongoing longitudinal prospective cohort study investigating cardiometabolic disorders in psychiatric patients.

**Results:**

Aside from well-known clinical risk factors for metabolic worsening (e.g. young age, first episode status, rapid weight gain during the first month of treatment and/or low initial BMI), additional risk factors have been recently identified. We showed an inverse association between socio-economic status (SES) and worsening of cardiometabolic parameters, adult patients with a low SES having a three-fold higher risk of developing metabolic syndrome over one year versus patients with a high SES (n=366). In addition, a causal inverse effect of educational attainment on BMI was revealed using Mendelian randomization in the UKBiobank (n=30’069). Results from an epigenome-wide association study (EWAS) performed in 78 patients before and after one month of treatment and from a genome-wide association study (GWAS) in 1924 patients will also be presented.

**Conclusions:**

Differences in clinical, genetic and environmental factors contribute to the differences in weight gain and metabolic disorders induced by psychotropic drugs. When starting a psychotropic drug at risk, a prospective monitoring of clinical (e.g. weight and blood pressure) and biochemical (fasting glucose, lipid levels) parameters is essential.

**Disclosure:**

Prof. Eap received honoraria for conferences or teaching CME courses from Janssen-Cilag, Lundbeck, Otsuka, Sandoz, Servier, Sunovion, Vifor-Pharma, and Zeller in the past 3 years. The other authors report no potential conflicts of interest. This work has

